# Torkildsen’s Ventriculocisternostomy First Applications: The Anthropological Evidence of a Young Slavic Soldier Who Died in the Torre Tresca Concentration Camp (Bari, Italy) in 1946

**DOI:** 10.3390/biology10121231

**Published:** 2021-11-25

**Authors:** Sara Sablone, Massimo Gallieni, Alessia Leggio, Gerardo Cazzato, Pasquale Puzo, Valeria Santoro, Francesco Introna, Antonio De Donno

**Affiliations:** 1 Section of Legal Medicine, Department of Interdisciplinary Medicine, University of Bari, Piazza Giulio Cesare, 11, 70124 Bari, Italy; alessialeggio@hotmail.it (A.L.); valeria.santoro07@gmail.com (V.S.); francesco.introna@uniba.it (F.I.); antonio.dedonno@uniba.it (A.D.D.); 2 Department of Neurosurgery, International Neuroscience Institute, Rudolf Pichlmayr Str. 4, 30625 Hannover, Germany; gallieni@ini-hannover.de; 3 Section of Pathology, Department of Emergency and Organ Transplantation (DETO), University of Bari “Aldo Moro”, 70124 Bari, Italy; gerardo.cazzato@uniba.it; 4 Section of Ophthalmology, Department of Medical Science, Neuroscience and Sense Organs, Bari Policlinico Hospital, University of Bari, 70124 Bari, Italy; pasquale.puzo@policlinico.ba.it

**Keywords:** forensic anthropology, Torkildsen’s shunt, Dandy’s point, neurosurgery, skeletal remains, World War II

## Abstract

**Simple Summary:**

Forensic anthropology deals with human skeletal remains for law and humanitarian purposes, and it is crucial in determining identity, interpreting traumas, and estimating time since death. Although it adopts a wide array of methods from many disciplines, the macroscopic observation of skeleton morphological characteristics is sometimes exhaustive for differential diagnosis between malformations, degenerative diseases, post-traumatic or iatrogenic lesions. In some cases, as described in this study, skeletal remains show signs of specific surgical techniques, so characteristic as to reconstruct almost faithfully the pathological history of the individual to whom they belonged and the therapeutic procedures the subject underwent. It is of even more interest if considering that, based on the time of death, the subject was among the first individuals who underwent an innovative surgical technique that would have revolutionized the surgical approach to a disease until then incurable. In these rare cases, skeletal remains become the historical testimony of surgery evolution, showing the traces of how men have over time perfected the medical treatment of their fellows.

**Abstract:**

Human skeletal remains are considered as real biological archives of each subject’s life. Generally, traumas, wounds, surgical interventions, and many human pathologies suffered in life leave identifiable marks on the skeleton, and their correct interpretation is possible only through a meticulous anthropological investigation of skeletal remains. The study here presented concerns the analysis of a young Slavic soldier’s skeleton who died, after his imprisonment, in the concentration camp of Torre Tresca (Bari, Italy), during the Second World War (1946). In particular, the skull exhibited signs of surgical activity on the posterior cranial fossa and the parieto-occipital bones. They could be attributed to surgical procedures performed at different times, showing various degrees of bone edge remodeling. Overall, it was possible to correlate the surgical outcomes highlighted on the skull to the Torkildsen’s ventriculocisternostomy (VCS), the first clinically successful shunt for cerebrospinal fluid (CSF) diversion in hydrocephalus, which gained widespread use in the 1940s. For this reason, the skeleton we examined represents a rare, precious, and historical testimony of an emerging and revolutionary neurosurgical technique, which differed from other operations for treating hydrocephalus before the Second World War and was internationally recognized as an efficient procedure before the introduction of extracranial shunts.

## 1. Introduction

The skeleton is a true biological archive because it faithfully testifies an individual’s state of health and relationship with the environment.

Surgical procedures undergone in life, autopsies and anatomical preparations/dissection can all leave clearly identifiable marks on human skeletal remains [1]. However, the distinction between them for scientific research can be challenging. For these reasons, it is firstly very important to distinguish ante-mortem interventions from post-mortem ones [2,3].

Among ante-mortem interventions, the trepanation of the skull arouses a particular forensic and anthropological interest, being regarded as one of the oldest known surgical procedures and having been reported in numerous prehistoric and archaic societies [4,5]. A thorough differential diagnosis is essential in these cases, especially in a skull lacking medical history or coming from an archaeological context where there is no other evidence that such operations were performed. Thus, identification of unhealed drilling (identifiable in cases where death has occurred soon after the procedure) is relatively straightforward, since tool marks provide direct evidence of surgical intervention. Conversely, a confident diagnosis is more difficult in healed defects of the skull, where bone remodeling that occurred during the survival time may hide the original surgical marks [6,7,8].

Exceptionally, however, anthropological investigations of human skeletal remains allow us to highlight signs of surgical procedures whose morphological pattern is so characteristic that it can be precisely attributable to specific techniques.

Here we describe the skeletal remains of a Slavic soldier who died at the age of 21 years, whose skull shows signs of antemortem neurosurgery procedures morphologically comparable to the Torkildsen’s ventriculocisternostomy (VCS) [9]. The skeletonized individual (stored in a coffin marked with the number 33 and bearing the years of birth and death “1925–1946”) is part of a skeleton collection of Slavic troops who had been detained and died in the prison camp of Torre Tresca (Bari Italy) during the Second World War, and whose remains were found in the Ossuary of the Monumental Cemetery of Bari [10,11].

An anthropological analysis was carried out to reconstruct the biological profile of the subject, and to describe the evident neurosurgical intervention performed on the skull.

## 2. Materials and Methods

**Figure 1:**
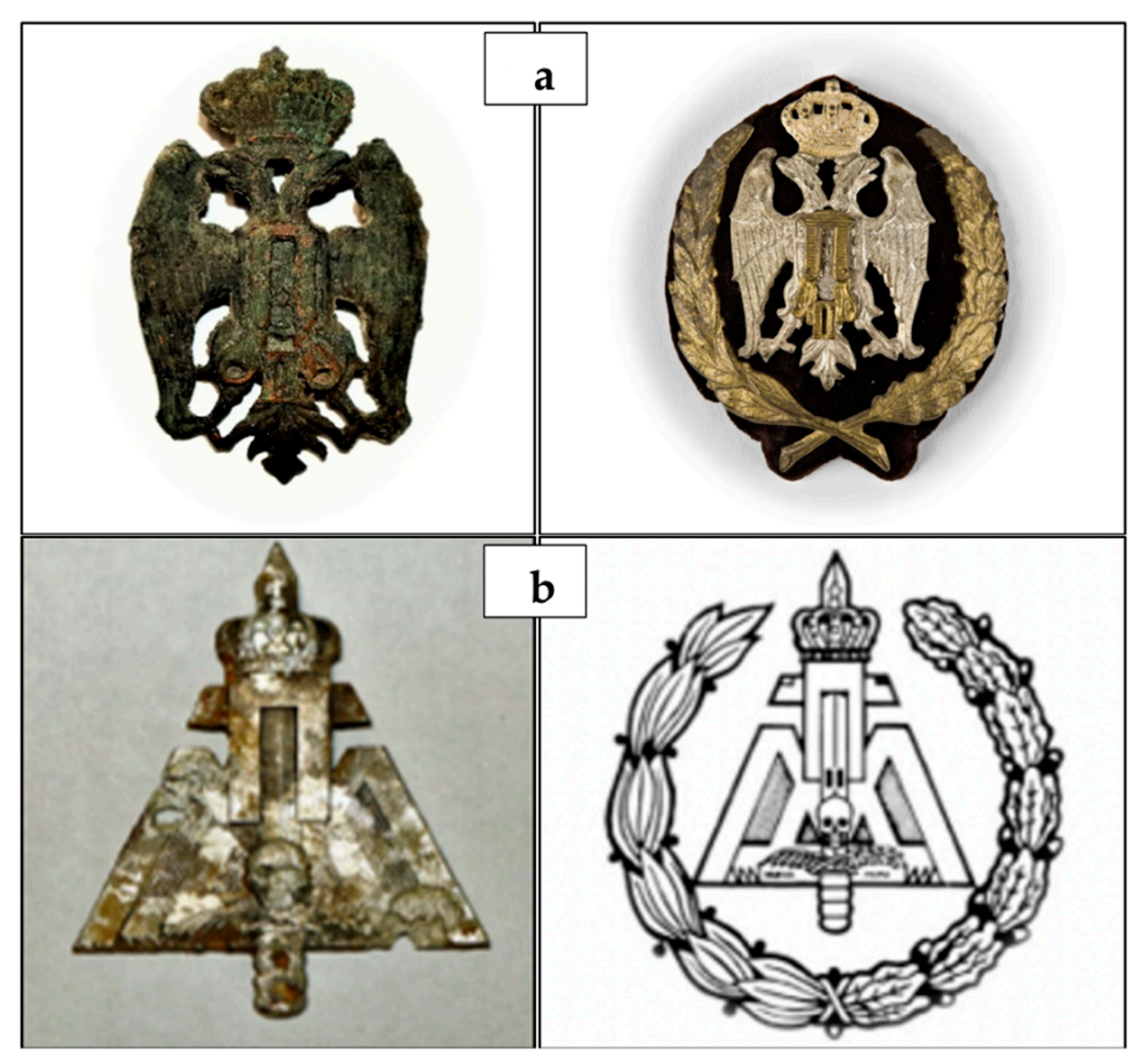
Objects placed in coffin No. 33. Frieze of the Royal Yugoslav Army, bearing the monogram of Peter II Karađorđević (**a**), and military badge of Ravnogorksi, representing the movement’s struggle of Ravna Gora under the leadership of General Dragoljub Draža Mihailović (**b**).

The examined subject is one of the 93 Slavic soldier skeletal remains from the Ossuary of the Monumental Cemetery of Bari. Several historical documents and the objects placed in the coffins allowed us to establish that the skeletons belonged to soldiers of the Yugoslav-Chetnik Royal Army, who were exiled to Italy in 1941 following the Yugoslavian invasion by the Axis powers. In particular, near the Slavic soldier skeletal remains contained in the coffin numbered 33, we found the frieze of the Royal Yugoslav Army, bearing the monogram of Peter II Karađorđević (last King of Yugoslavia between 1934 and 1945), which soldiers usually wore on a pointed sheepskin hat. Moreover, we found the military badge of Ravnogorksi, representing the movement’s struggle of Ravna Gora, where the Slavic troops to whom the skeletal collection belongs, were under General Dragoljub Draža Mihailović’s leadership (Figure 1).

The investigations were carried out in the Laboratory of Forensic Anthropology of the Department of Forensic Medicine of Bari. The skeleton was almost completely intact. Physical anthropological methods were applied to macroscopically identify human remains and to reconstruct the biological profile [12]. In particular, morphological parameters were used for the skull (complete with mandible) and the hip (fully preserved) for sex estimation [13]. Age at death determination has been carried out by analyzing the epiphyseal fusion degree [14] and based on Nemeskéri et al.’s method [15]. Moreover, the study of the degree of dental wear and the study of the morphology of the pubic symphysis and of the ilium auricular surface were applied [16,17,18,19]. As a metopic suture was detected, cranial suture fusion degree has not been considered for age-at-death evaluation [20]. Trotter’s and Gleser’s methods were used to determine stature [21]. Anthropometric indices were calculated to determine the bone robustness of the subject [22].

Although research can count on many technological and innovative techniques for improving the interpretation of skeletal lesions (such as microscopy, imaging and chemistry), paleopathological and traumatic injury detection was conducted according to the most common anthropological analyses carried out, which are still essentially based on macroscopic morphological observation [23,24]. Histological and immunohistochemistry investigations, useful for the age estimation of bone lesions [23,25], were not carried out to avoid the partial destruction of the skeleton.

## 3. Results

The skeleton under examination belonged to a man with an age-at-death of 20–25 years, height 174.4 ± 3.3 cm. Osteometric measurements indicated a mesocranial skull, with a horizontal cranial index of 75.0–79.0. The presence of the metopic suture was detected. The platimeric index of the femur, with a value of 103, indicated stenomeria, suggesting a poor development of trochanters. The robustness index calculation resulted in a value of 11, indicating a weak femur, while the pilastric index was equal to 75, suggesting a weak activity of the thigh muscle of the subject in life. The cnemic index of the tibia, with a value of 134, indicated eurychnemia, which is suggestive of poor muscular development (calf muscle).

During paleopathological analyses, a cribra orbitalia of severity 3 and healing grade 1 was found on both the left and right orbital roof [26], suggesting that the subject probably suffered an iron deficiency, or infectious disease, or scurvy, or B12 deficiency megaloblastic anemia [27], or that he developed a hematological disease causing a hemopoietic marrow expansion [28].

**Figure 2:**
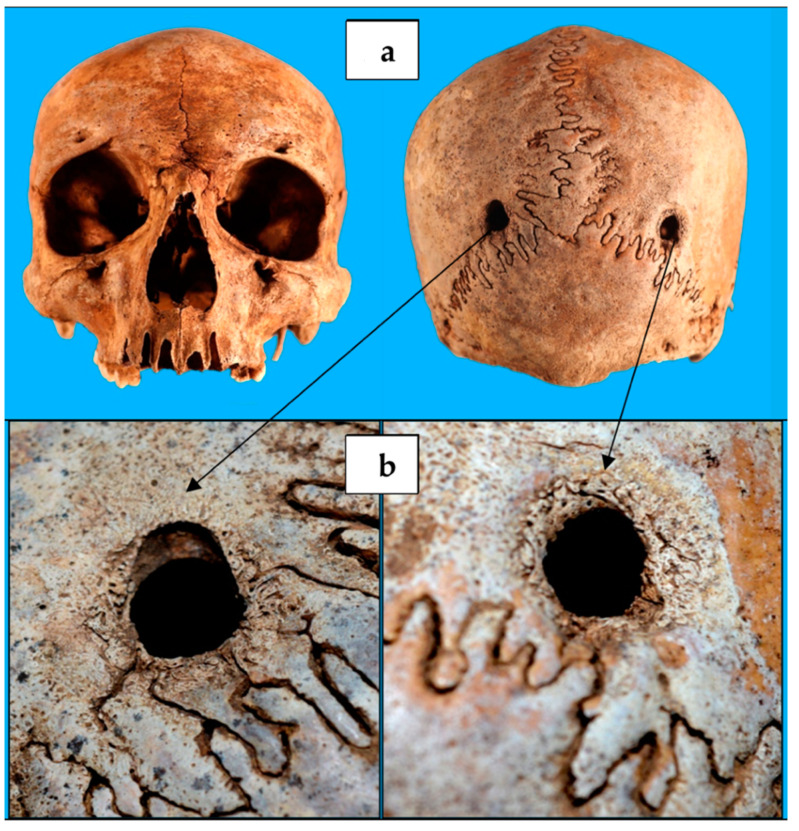
Cranium of the individual contained in coffin No. 33. (**a**) The two circular-shaped lesions of parietal bones, (**b**) with evidence of a healing process along their edges.

Investigation for any traumatic lesions revealed the presence of two cranial circular holes (Figure 2), each with a diameter of 1 cm, and located at the level of the parietal bones, near the meeting point between the sagittal suture and the lambdoid one. They were specularly positioned along an almost transversal axis, and they had margins with traces of bone remodeling, allowing us to establish that the two holes’ production occurred antemortem [23,29,30].

**Figure 3:**
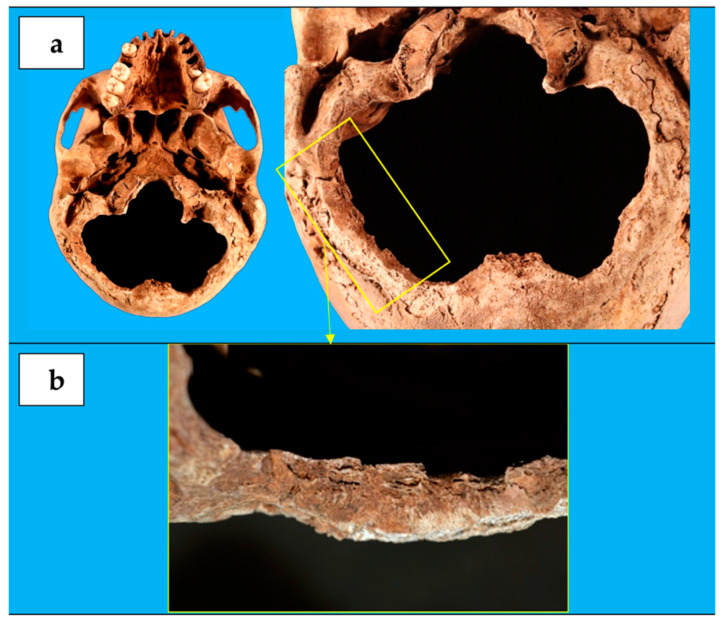
(**a**) The clover-shaped loss of substance in the occipital bone lower portion, (**b**) with cutting blade marks over part of the lesion edges and poorly evident healing process.

The most significant skull lesion consisted in a loss of substance in the occipital bone lower portion (Figure 3), resulting in an occipital foramen enlargement. It was clover-shaped, with a transverse diameter of 10 cm and an antero-posterior diameter of 8 cm. Its margins run along the occipito-mastoid sutures bilaterally and between the upper nuchal line and the lower one at the rear, lapping the outer occipital protuberance. Typical cutting blade marks were still observable over part of the lesion edges, showing a poorly evident healing process and suggesting that the lesion had been probably produced later than the paralambdoid ones [2].

## 4. Discussion

The first visual examination of the soldier’s skull reveals two holes in the posterior surface of the parietal bone, located above the lambdoid suture. When related to the subject’s occupation, the two cranial holes were initially considered as the result of gunshots. However, the “bullet theory” was ruled out firstly because of the presence of bone granulation signs, which are a vital reaction demonstrating the soldier didn’t die after the holes’ production. Even assuming the subject survived any gunshot wounds, this theory would still be unreliable because of the skull holes’ symmetry, the absence of chipping and/or radial fractures, the absence of exit wounds and of surgical accesses used to remove bullets or to treat gunshot-related brain lesions [23].

Another characteristic relief is the lack of the occipital squama lower portion, producing a foramen magnum abnormal dilatation. Because of the symmetry of bony edges and the absence of radial or concentric fractures (which are frequent in case of blunt trauma from large impacting surfaces), for this relief we also ruled out the post-traumatic origin [23].

All these elements lead us to suppose that the subject underwent a craniectomy or a decompressive craniotomy in life.

In order to make diagnostic hypotheses as reliable as possible, it has been rightly noted that the two parietal holes were located at the so-called “Dandy’s points” (named after a famous pioneer of neurosurgery: Walter Dandy). This point, identifiable 2 cm sideways to the rear midline and 3 cm below the inion, was described in the early decades of the 1900s to carry out a diagnostic procedure called cerebral ventriculography, useful for detecting hydrocephalus but also intracranial diseases (such as tumors) resulting in abnormal CSF circulation [31,32]. Thus, we speculated that the soldier suffered in life from hydrocephalus. As a differential diagnosis, we included brain tumors, or Chiari malformation, or a post-traumatic hemorrhagic pathology, which in some cases are themselves the cause of hydrocephalus.

The Dandy’s point was (and still is in selected cases) used to easily reach the occipital horn of the lateral ventricle and thus to insert a drainage catheter [33]. Cerebral ventriculography (or pneumoencephalography), as originally described by Dandy, required the insertion in the lateral ventricles of a needle connected by a double valve to a syringe, allowing CSF to be suctioned and air to be introduced at the same time in order to perform an X-ray image.

During childhood, this procedure was carried out by inserting the needle directly into the anterior fontanelle [34]. With its physiological closure, the procedure was performed by drilling a small hole at the Dandy’s point [33,34]. It is consistent with our findings, since the soldier’s skull shows no signs of dilatation or deformation, suggesting that he suffered from an intracranial disease at an age when he had already completed the skull bones’ fusion. For the same reason, we ruled out congenital/developmental lesions from the etiological hypotheses of hydrocephalus, such as Chiari malformation or skull-cervical hinge anomalies [8].

Moreover, an in-depth analysis of the two paralambdoid cranial holes shows some differences: the left hole coincided with the Dandy’s point, while the right one was at least 1 cm more lateral. This hole may have led to an unsatisfactory trajectory to find the occipital horn of the right ventricle and to collect CSF. Therefore, it may have been not used, undergoing bone edges’ remodeling. Conversely, the left hole has sharper margins in the upper portion, suggesting that it was probably done later or that it was catheterized or drilled more than once [23].

Based on skeletal findings, the most reasonable hypothesis is that the subject firstly underwent diagnostic ventriculography and, subsequently, a suboccipital craniotomy to decompress the posterior cranial fossa. If the subject had been suffering from hydrocephalus, this could have been treated with a Torkildsen’s shunt [35,36], whose technique is consistent with our findings on the soldier’s skull.

Arne Torkildsen was a pioneering Norwegian neurosurgeon, the first to perform (on September 9, 1937) a clinically successful procedure, called VCS, for shunting CSF in non-communicating hydrocephalus [9,31,35]. The surgical method consisted in a burr hole placed for ventriculography via one of the occipital horns of the lateral ventricle. Then a suboccipital craniotomy was performed and a rubber tube was connected between the occipital horn and the cisterna magna. A groove was chiseled in the skull underneath the scalp just wide enough to hold the catheter. The procedure was performed under local anesthesia [9,32,37]. The technique soon received international acceptance as a standard procedure for treating obstruction of the aqueduct/third ventricle, and it gained widespread use in the 1940s, before extracranial shunts’ introduction [9,38].

Skull morphological analyses did not allow us to precisely establish what neuropathology required the above-mentioned neurosurgical interventions. It was only possible to reasonably rule out congenital/developmental anomalies. However, because of the symmetry and the highly suggestive morphological pattern of the skull defects highlighted in this study, it is very unlikely that they could have been directly produced by such indefinable pathologies (e.g., infection, tumors, bone seeking metastatic malignancies) or accidental/traumatic events, as compared to surgery. Moreover, based on the absence of other trepanned skulls among the examined Slavic troop skeletal remains, ante-mortem symbolic and/or magico-ritual cranium drilling could also be excluded [8].

In the present study, the lack of histological and immunohistochemical investigations, as well as of radiological analyses (which are complementary to the former), did not allow post-traumatic interval estimation [25,39]. However, the timing of a specific response directly on dry and skeletal remains is still unexplored [23]; thus, defining the time elapsed since the skull lesions were produced becomes a difficult task.

What we can reasonably affirm is that the skull defects highlighted in the present study are ante-mortem lesions. Moreover, their morphological assimilation to newly implemented neurosurgical procedures allows dating them a few years before the soldier’s death.

Following Torkildsen’s shunt, the subject may have been able to return to a roughly normal lifestyle, to be enrolled in the army, and to participate in the Second World War events, which unfortunately led to his death in a concentration camp in Italy.

## 5. Conclusions

The 20th century saw the worldwide implementation of neurosurgical procedures such as ventriculography and VCS. Certain controversies persist in skull trepanation differential diagnosis, which could be very challenging for forensic anthropologists and pathologists. Although unusual, signs of specific surgical techniques may be found on human skeletal remains, so characteristic as to reconstruct almost faithfully the clinical and pathological history of the individual to whom they belonged.

The human skeleton examined is the unique anthropological evidence of a Torkildsen’s VCS performed on a Slavic man in the 1930s–1940s. Because of the above-mentioned limitations in differential diagnosis, it is not possible to precisely establish which was the neuropathology affecting the subject. However, based on historical evidence suggesting that the man was a soldier, it may be possibly assumed that he had successfully undergone the VCS. The procedure, first performed in 1937, may have been carried out on the man shortly before the onset of the Second World War. The man’s subsequent enrollment as a soldier led him to die, probably of other causes, in the Torre Tresca (Bari) concentration camp, after his deportation to Italy in 1946.

## Data Availability

Not applicable.
